# Epigenetic Therapy as a Putative Molecular Target to Modulate B Cell Biology and Behavior in the Context of Immunological Disorders

**DOI:** 10.1155/2020/1589191

**Published:** 2020-02-08

**Authors:** Thayse Pinheiro da Costa, Marcia Cury El-Cheikh, Katia Carneiro

**Affiliations:** Federal University of Rio de Janeiro, Institute of Biological Sciences, Laboratory of Cell Proliferation and Differentiation, Av. Carlos Chagas Filho 373 Room F2-01: 21941-902, Brazil

## Abstract

Histone Deacetylase- (HDAC-) dependent epigenetic mechanisms have been widely explored in the last decade in different types of malignancies in preclinical studies. This effort led to the discovery and development of a range of new HDAC inhibitors (iHDAC) with different chemical properties and selective abilities. In fact, hematological malignancies were the first ones to have new iHDACs approved for clinical use, such as Vorinostat and Romidepsin for cutaneous T cell lymphoma and panobinostat for multiple myeloma. Besides these promising already approved iHDACs, we highlight a range of studies focusing on the HDAC-dependent epigenetic control of B cell development, behavior, and/or function. Here, we highlight 21 iHDACs which have been studied in the literature in the context of B cell development and/or dysfunction mostly focused on B cell lymphomagenesis. Regardless, we have identified 55 clinical trials using 6 out of 21 iHDACs to approach their putative roles on B cell malignancies; none of them focuses on peritoneal B cell populations. Since cells belonging to this peculiar body compartment, named B1 cells, may contribute to the development of autoimmune pathologies, such as lupus, a better understanding of the HDAC-dependent epigenetic mechanisms that control its biology and behavior might shed light on iHDAC use to manage these immunological dysfunctions. In this sense, iHDACs might emerge as a promising new approach for translational studies in this field. In this review, we discuss a putative role of iHDACs in the modulation of peritoneal B cell subpopulation's balance as well as their role as therapeutic agents in the context of chronic diseases mediated by peritoneal B cells.

## 1. Introduction

### 1.1. Peritoneal Cavity and Its Cellular Subpopulations

The peritoneal cavity (PerC) is a singular compartment where cells of the immune system involved with innate immunity reside immersed in the peritoneal fluid and in histological organizations highly reactive as the mesentery and the omentum [1–6]. The peritoneum is a serous membrane composed of mesothelial cells, named parietal and visceral peritoneum, which cover the cavity and most of the abdominal organs [7–9]. Thus, the PerC is a dynamic structure that selectively attracts and maintains specialized cells travelling between fluid and adjacent tissues, mesentery and omentum. Both mesentery and omentum contain “milk spots” (MSs) that are organized as loose collections mainly composed of monocytes and lymphocytes, which are involved by adipose tissues and a mesothelial layer [6, 10–14]. The fenestrations present in the mesothelial layer are permissive to the flow of cells back and forth once the MSs lack the afferent lymphatic vessels. This configuration of fenestrations, or stomata-like structures, is considered to promptly regulate the volume of fluid as well as the mobilization of defense cells, maintaining homeostasis [6, 8, 15].

On the other hand, through the diaphragmatic lymphatic vessels, the lymphocytes in the peritoneal fluid can gain the systemic circulation and come back to MSs that are formed around a glomerulus-like knot of blood vessels [10, 11]. Through the high endothelial venule (HEV) expressing addressins, essential for “ecotaxis” [16] or “homing” [17], these cells can achieve the tissues contributing, in this way, to the diversity of cells in the peritoneum [6, 10, 11].

### 1.2. Peritoneal Cell Populations

#### 1.2.1. Monocytes and Macrophages

The peritoneal cavity is a singular compartment in which cells of the immune system reside and interact, being similar to the secondary lymphoid organs, but without presenting the organized histological distribution which is typically found in these organs. Under physiological conditions, the peritoneal cellular population is mostly composed of monocytes, macrophages, and B cells. In addition, T cells, NK (natural killers) cells, dendritic cells, and granulocytes can also be found [18]. Peritoneal macrophages are among the best-studied macrophage subsets since they play important roles in the control of infections and a range of pathologies. In fact, Ghosn and colleagues defined two subsets of macrophages that coexist in the peritoneal cavity: the large peritoneal macrophage (LPM) and the small peritoneal macrophage (SPM) [19]. SPMs and LPMs exhibit specialized functions, since SPMs display a proinflammatory profile and LPMs appear to play a role in maintaining physiological conditions. In addition, LPMs are required to stimulate the production of immunoglobulin A (IgA) by peritoneal B1 cells in a retinoic acid-dependent fashion [18]. Thus, the interactions between the different subsets of macrophages and other populations of the peritoneal cavity appear to play a crucial role in the immune status of this anatomic site.

#### 1.2.2. B Lymphocytes

Approximately 40% of the peritoneal cavity cells are B lymphocytes which are subdivided into B2 (conventional B cell) and B1 cells. B2 cells are part of the adaptive immune response characterized by the production of high-affinity and isotype-switched antibodies. B1 cells arise early during ontogeny becoming a self-renewing cell population that promptly responds to several stimuli secreting low affinity, polyreactive, and natural IgM antibodies, composing along with macrophages the first line of an organism's defense [20, 21]. Besides the functional characteristics, B1 cells are distinguished from B2 by the surface phenotype once they express high levels of IgM and low levels of IgD, CD23, and B220. They are also Mac-1-positive, and part of them is known as B1a cells that express CD5, whereas B1b cells are CD5-negative [20, 22, 23].

#### 1.2.3. B1 Cell Behavior and Biology

B1 lymphocytes are characterized by the ability to switch from IgM to IgA secretion faster than follicular spleen cells or peritoneal B2 lymphocytes if properly stimulated [24]. Similar to B2 lymphocytes, B1 cells differentiate into plasma cells by a B lymphocyte-induced maturation protein-1- (Blimp-1-) dependent pathway, a master regulator that is constitutively expressed by these cells and may contribute to explain the continuous and spontaneous secretion of IgM [25]. The immediate response of these cells to a given stimuli may lead to an efficient exit from the peritoneal cavity. In accordance, B1 cells may modulate their adhesion molecules and differentiate into plasma cells still in the peritoneal cavity. On the other hand, these cells can adhere to mesenteric membranes or omentum, as described in the literature, if previously activated by the absence of galectin-3 [26].

The important self-renewing property of B1 cells can be explained, at least in part, by the finding that they constitutively express activated nucleic acid transducer (STAT3), which may play a role in the regulation of cyclin D2, contributing in this way to the proliferative behavior of these cells [27]. The maintenance of this cell population by self-renewal appears to be important for antibody production, since B1 cells secrete significant amounts of IgM/IgA and are considered to play an important role in natural immunity [23]. Among these Igs secreted by B1 cells, it is worth mentioning the secretion of 50% of the IgA present in the lamina propria of the intestine, while the remaining IgA secretion is due to the conventional B cells found in Peyer's plaque. Such characteristics significantly contribute to the defense against pathogens found in the enteric tract [28].

In addition to the constitutive expression of STAT3 by B1 cells, and in contrast to B2 cells, these cells also express the interleukin-5 receptor alpha (IL-R5*α*) chain, which renders to this subset the ability to specifically respond to interleukin 5 (IL-5), GM-CSF, and IL-3 [29]. In this way, B1 lymphocytes can be maintained *in vitro* if properly stimulated by IL-5 allowing the analysis of their behavior such as the proliferation rate, survival, and/or differentiation into Ig-producing cells and IgA switch under experimental conditions [26, 30, 31].

It is noteworthy that the constitutive expression of both STAT3 and IL-R5*α* enhances the responsiveness of B1 lymphocytes with regard to the balance between proliferation and differentiation, which may in turn induce cell transformation or the production of a self-reactive Ig repertoire. In this context, the main source of IL-5 and IL-3 in the peritoneal cavity is due to resident mast cells that mostly contribute to B1 cell biology, controlling its proliferation and differentiation.

The identity of B1 cells in the human system remains poorly defined. Recently, functional criteria were established on the basis of the murine B1 cell behavior, such as spontaneous antibody secretion and production of autoreactive antibodies [32, 33]. The human B1 cells display phenotypic markers such as CD20^+^, CD27^+^, CD43^+^, and CD70^−^ in both umbilical cord blood and adult peripheral blood.

In contrast to mice, human B1 cells are found in high frequency in peripheral blood instead of the peritoneal and pleural cavities. As the human B1-like cells are increased in patients with autoimmune diseases, they could be a rich source of autoantibodies and a putative T cell activity modulator. In this context, manipulation of human B1 cells may be a good target to target autoimmune diseases and other immune dyscrasias.

## 2. Epigenetic Mechanisms and B Cell Plasticity and Behavior

### 2.1. Epigenetic Mechanisms

Epigenetics refers to the set of mechanisms that controls the gene expression through chromatin remodeling events and chromatin status that, in turn, can be stably inherited through generations in the absence of changes or mutations in the DNA sequence. Such chromatin status includes DNA methylation, posttranslational histone modifications, and RNAi [34]. The organization of chromatin in functional units is the central component of the epigenetic regulation. Chromatin is composed of DNA plus proteins, and its functional unit is the nucleosome, a structure made up of 147 pb of DNA wrapped around a histone octamer. The most common histone octamer is composed of 1 dimer of each histone H2A, H2B, H3, and H4 which are linked to one another by a H1-binding histone, forming a highly compacted DNA structure [35]. However, the nucleosome is not simply a tool for packaging DNA and decreasing the space it occupies in the nucleus of the cell, but it is mainly a dynamic structure that perfectly mirrors the gene expression pattern of the cell [36].

### 2.2. Histone Posttranslational Modification and Histone Deacetylase Activity

As a general rule, transcriptionally active chromatin regions are hyperacetylated, unlike heterochromatin regions that are hypoacetylated and methylated at CpG islands [35].

Histone acetylation is one of the most well-characterized PTMs and is a key player during chromatin remodeling and gene transcription [37]. Histone acetylation levels are the result of the balance between the opposing activities of the Histone Acetyltransferase (HAT) and Histone Deacetylase (HDAC) enzymes. HATs catalyze the addition of acetyl to specific lysine residues of the histone N-terminal tail while HDACs catalyze the removal of the acetyl group and are tightly related to transcriptional repression [38].

The enzyme first reported displaying HDAC activity was cloned from yeast and was named Rpd3 [39]. Next, other enzymes with HDAC activity were cloned and classified according to their homology to the yeast Rpd3. To date, 18 HDACs have been identified in mammals, and these have been grouped into 4 different classes [40]: class I HDACs (HDACs 1, 2, 3, and 8) [41], class II HDACs (HDACs 4, 5, 6, 7, 9, and 10) [42], class III HDACs, also called sirtuins (SIRT 1, 2, 3, 4, 5, 6, and 7) [43], and class IV HDAC that is represented only by HDAC 11, and little is known about this enzyme.

Histone acetylation takes place at the amino group of different lysine residues, located in the N-terminal tail of different types of histones. Regardless of the fact that all histones are acetylated *in vivo*, the acetylation of histones H3 and H4 is better characterized than the acetylation of histones H2A and H2B. Very well-known histone acetylated lysine residues are K9 and 14 on histone H3 and K5, K8, K12, and K16 on histone H4 [44]. The HDAC active site consists of a charge relay system in which the presence of Zn^2+^ cofactor is essential for its deacetylase activity [45]. The action of HDACs generates a hypoacetylation status resulting in chromatin compaction and transcriptional repression [46].

### 2.3. iHDACs

An important strategy for studying HDACs is employing strategies to pharmacologically knock down their enzymatic activity. This strategy enables to evaluate the role of the enzyme in very specific biological contexts since the effects evoked by iHDACs are mostly reversible and selective [45]. iHDACs will promote chromatin hyperacetylation, which is associated with chromatin remodeling to a looser and more accessible state favoring gene transcription [47]. However, the cellular response to iHDACs is complex because nonhistone proteins may also hyperacetylate [47].

iHDAC can be found as natural or synthetic compounds that target the classic enzymes of the HDAC family. They are distributed in different classes of chemical compounds, including hydroxamic acids, carboxylic acids, benzamides, and cyclic tetrapeptides [48]. The overall mechanism of inhibition of the HDAC enzymatic activity is similar among the drugs, which fit the active site of the enzyme and act as zinc ion chelants. Thus, iHDACs block the access to the active site of HDACs and prevent them from binding to the acetylated lysine substrate [49]. Most HDAC inhibitors block in a nonspecific fashion enzymes belonging to classes I and II, and these are named HDAC pan-inhibitors. However, since HDAC inhibitors have different structures, there is also a difference in the efficiency and specificity of iHDACs [50] (Table 1).

iHDAC inhibitors transiently block HDAC activity and represent a promising class of anticancer agents. Four of iHDACs have been approved by the US Food and Drug Administration: Vorinostat, Romidepsin, Belinostat, and panobinostat. All of them have been approved for use in the treatment of hematological tumors, such as Hodgkin's lymphoma [51] (Table 1). Other iHDACs are in different stages of clinical trials for several hematological and solid tumors, such as Entinostat and Valproic acid, the latter being already used in the treatment of epilepsy and bipolar disorders [52].

In particular, Trichostatin A (TSA) is one of the most potent and specific iHDAC to class I and II HDACs [53]. TSA is a natural compound obtained from *Streptomyces*. This is a reversible inhibitor of HDAC activity belonging to the group of hydroxamic acids [54]. TSA has been shown to disrupt gene expression in tumor cells and has been shown to be a good therapeutic agent in several other diseases, such as asthma [55]. Currently, the use of TSA as a therapeutic agent has been neglected due to the high costs for large-scale production. However, TSA is used as a reference substance in the research for the development of new iHDACs [56].

Among the most relevant biological functions of iHDACs, we highlight their effects on cell death and differentiation in addition to cell cycle blockade in transformed cells [57] (Table 1). iHDACs are well tolerated in clinical protocols and are not as effective in solid tumors as in hematological cancers. Interestingly, healthy cells are resistant to cell death evoked by iHDACs and rapidly reverse the adverse effects of iHDACs [58]. Recently, clinical studies using iHDACs have been extended to a wide range of nontumoral diseases such as anemia, HIV infections, neurodegenerative diseases, and inflammatory disorders. It is believed that these drugs induce transcriptional reprogramming and therefore have extensive therapeutic benefits [48].

### 2.4. HDACs and B Cells

The hematopoietic system, in particular lymphopoiesis, is composed of several decision-making points for cell fate acquisition which are characterized by the expression of a set of genes that are lineage specific. The expression of such genes in progenitor cells must be tightly controlled in order to give rise to precise patterns of gene expression profiles in a dynamic fashion. Such dynamics and precision are mostly conferred by the epigenetic machinery down the differentiation cascade [59]. In this sense, histone acetylation and deacetylation can shape the pattern of gene expression in response to environmental clues associated with the B cell differentiation cascade. The master gene Pax5 is an essential transcription factor necessary for the maintenance of B cell fate. Molecular studies on pro-B cells revealed that Pax5-activated genes display highly acetylated lysine residues on histone H3 [60]. In deficient cells for Pax5, the levels of histone acetylation are dramatically reduced or lost, indicating that this transcription factor is essential for chromatin remodeling during B cell fate acquisition.

In fact, it has been shown that the expression of Aicda and Prdm1, two key genes for B cell differentiation, occurs upon epigenetic changes on promoter regions. Activation-induced cytidine deaminase (AID) is encoded by the Aicda gene, which is expressed in a stage-specific manner during B cell development [61]. This protein is necessary for class-shifting recombination (CSR) and somatic hypermutation (SHM), a critical event that leads to the production of protective antibodies against microbial pathogens. Hypermutated and class-shifted B cells have also differentiated into antibody-secreting plasmocytes in a Blimp-1-dependent manner, which is encoded by the Prdm1 gene [62]. The silencing of the Aicda and Prdm1 genes by iHDAC has been found to be intrinsic to spleen B cells and independent of other cellular elements and is associated with a concomitant increase of microRNAs followed by downregulation in the expression of these genes [63]. As a consequence of the inhibition of Aicda and Blimp-1 expression, there is a decrease in CSR and SHM in antibody responses and B lymphocytes remain IgM^+^, leading to an increase in Ig levels.

One of the nonhistone proteins which is also substrate for HDACs is STAT3, which upon acetylation on a lysine residue becomes transcriptionally active [64]. Considering that STAT3 activation promotes cell cycle progression, cell survival, and proliferation, HDACs can emerge as putative targets to control B cell behavior. In fact, the treatment of dendritic cells and tumoral cell line with HDAC inhibitors (iHDACs) has shown an increase in the acetylation levels of STAT3 [65, 66]. However, there is a lack of studies on a putative role of iHDACs on B1 cell behavior and biology.

#### 2.4.1. Are iHDACs Putative Therapeutic Targets for Autoimmune Diseases?

Although a correlation between HDAC-dependent epigenetic mechanisms and autoimmune disease has been explored in the literature, as described by Mazzone et al. [67], iHDAC effects on B cell biology and behavior and its role as putative targets for use in autoimmune diseases have been little explored in the literature. In fact, only 35 studies published in the last 5 years approached the role for iHDACs in the modulation of B lymphocyte behavior. As summarized in Table 1, it is possible to note that only 3 papers approached a putative role for iHDACs in the context of B cell-mediated autoimmunity (highlighted with asterisks in Table 1). For instance, it has been shown that bone marrow or spleen B cells from mice MRL/MpJ-Faslpr displaying lupus-like disease overexpress HDACs 6 and 9. In addition, HDAC activity blockade with the iHDAC ACY-738 resulted in bone marrow pre-B cell apoptosis through a Bax protein signaling cascade [68]. Another report, using the same experimental model, demonstrated that the iHDAC panobinostat (that targets class I, II, and IV HDACs) dramatically reduced the number of circulating B220^+^ CD19^+^ B cells coupled to the reduction of autoantibodies, glomerulonephritis, and interstitial nephritis [69]. Interestingly, panobinostat (LBH589) has been approved for use by the European Medicines Agency, and FDA has accelerated the approval for use in multiple myeloma (2015). In 2018, the iHDAC CDK-506 was shown to significantly decrease the levels of inflammatory mediators in NZB/W F1 mice, another model that mimics lupus [70]. Another interesting scenario for iHDAC as anti-inflammatory agents is in inflammatory bowel disease (IBD). In fact, two different HDAC 6 selective inhibitors, BML-281 [71] and LTB2 [72], were shown to attenuate dextran sulfate sodium- (DSS-) induced colitis in the mouse model. Taken together, these results indicate that iHDACs may provide beneficial effects modulating inflammatory and autoimmune signaling pathways.

Another interesting aspect of iHDACs in the context of B cell behavior and biology is the increasing interest of translational studies. In fact, we compiled 979 clinical trials testing 20 different iHDACs to properly explore their putative role as antitumoral agents (Table 1). Among them, we highlight 6 of them, which as a whole have been used in 55 clinical trials exclusively in the context of B cell lymphomas: Entinostat, panobinostat, Ricolinostat, Romidepsin, Vorinostat, and Valproic acid. Panobinostat is the only one that reduces autoimmunity antibody-producing cells and may emerge as a putative tool to properly control autoimmune diseases mediated by B cell (Table 1).

In Table 2, we summarized the papers that have investigated the molecular targets and mechanisms involved in HDAC activity in the context of B cell development and disease. In some cases, the molecular target of iHDACs involves cell cycle disruption and apoptosis induction through p21 and tumor growth suppression through Myc or p53 signaling pathway (Table 2). In addition, while most part of the 11 papers listed focuses on B cell malignancies, only 1 focused on lupus-like autoimmune disease. In this paper, the pattern of mRNA expression of HDACs 6, 9, and 10 was disrupted. While bone marrow B cells upregulated the expression of HDACs 9 and 6, HDAC 10 was downregulated. On the other hand, in splenic B cells, HDACs 6 and 10 were upregulated [68]. Taking into account that panobinostat is already in phase III of clinical trial and that it inhibits HDACs 6, 9, and 10, this iHDAC may emerge as a putative molecular tool to successfully modulate B cell behavior in the context of autoimmune diseases.

Thus, due to the lack of knowledge on iHDACs in the context of B cell dysfunction in autoimmune disease, a best comprehension of the epigenetic landscape associated with the development and differentiation of B cells is crucial to properly understand their behavior and biology. To better understand, the molecular mechanisms that cause or result from a disruption in the epigenetic landscape of B cells may aid us to design new strategies and therapeutical approaches to better handle a range of pathophysiologies associated with disruptions on B cell behavior.

**Figure 1:**
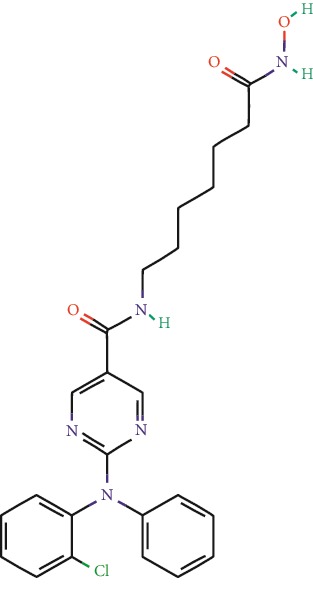
HDAC activity blockade disrupts the cell cycle in the peritoneal cavity. C57BL/6 male mice were injected with iHDAC (40 nM, gray bar, or 100 nM, white bar; Trichostatin A: TSA) in the peritoneal cavity, and cell cycle was analyzed upon 48 hours. The control group was injected with DMSO. Regardless of the fact that iHDAC did not impact on cell morphology ((a) control; (b) iHDAC (40 nM); (c) iHDAC (10 nM)), we detected cell cycle arrest in the G0/G1 phase. Data are represented as means ± SEM and are representative of 4 animals in each group. Red arrows show proliferative clusters.

In fact, regardless of the fact that different HDACs have been mechanistically implicated in B cell development and/or malignancy (Table 2), a putative role for HDAC activity on the biology and/or behavior of B1 cells, as well as on the maintenance of peritoneal homeostasis, has not yet been adequately addressed in the literature so far. To collect evidences to better address this issue, we asked whether iHDAC injection in the peritoneal cavity would impact on peritoneal cell behavior. For example, the injection of iHDAC into the peritoneal cavity of mice is able to disrupt the cell cycle of the total population in the peritoneal cavity, causing the cell cycle exit and cell resting in the G0/G1 phase (Figure 1).

**Figure 2:**
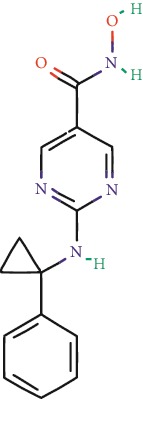
HDAC activity is necessary for B1 cell behavior. Peritoneal cells were harvested from C57BL/6 male mice and cultured in medium (RPMI), medium+IL-5 (RPMI+IL-5), medium+iHDAC (RPMI+iHDAC), or medium+IL-5+iHDAC (RPMI+IL-5+iHDAC). Upon 48 hours in cell-free culture (a–e) or 24 hours on a feeder layer (h–j), B220^+^ cells were quantified by flow cytometry and B1 cells (B220^low^/CD11b^+^) were phenotyped. The data are represented as means ± SEM and are representative of cell cultures of 3 distinct animals in each experimental condition. IL‐5 = 20 ng/mL; TSA = 40 nM.

This indicates that the HDAC activity blockade may emerge as an important strategy to modulate the behavior of B1 cells *in vivo*. In fact, upon cell culture *in vitro* in the presence of IL-5 and iHDAC, the B220^low^/CD11b^−^ subset was enriched after 48 hours of culture (Figures 2(a)–2(e)), indicating that HDAC activity is necessary for peritoneal homeostasis. Indeed, if a feeder layer, composed of adherent cells from the peritoneal cavity, was added to the culture, a specific increase in the B220^low^/CD11b^+^ subset was observed, suggesting that the inhibition of HDAC activity is also required for the behavior of subset of cells, such as macrophages, as previously demonstrated by our group [73]. In this sense, the blockade of HDAC activity could globally modulate the behavior of different cell populations in the peritoneal cavity and promote a feedback loop, which in turn is capable of promoting the expansion of the B220^low^/CD11b^+^ subset already upon 24 h of cell culture (Figures 2(a)–2(j)).

**Figure 3:**
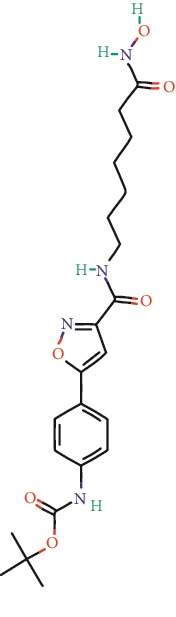
HDAC activity is necessary for peritoneal homeostasis and B cell function. C57BL/6 male mice were injected with iHDAC (TSA 100 nM) in the peritoneal cavity, and upon 5 days, peritoneal cells (a–c) and the mesenteric lamina (d–f) were analyzed. B1 cells were specifically increased in iHDAC-treated groups (c). The mesenteric lamina structure was disrupted, and degranulated mast cells were observed ((e, E′), red arrow). ELISA assay showed that while iHDAC-treated B cells secreted higher levels of IgM, the levels of IgA did not change under the same conditions. Degranulated mast cells were observed only in the presence of iHDAC (B′, E′). Data are represented as means ± SEM and are representative of 4 animals in each group. Proliferative cluster (red arrow (b)). ^∗∗^*P* = 0.0079.

In agreement, *ex vivo* phenotyping of the B population of the peritoneal cavity after iHDAC injection showed a specific increase of the B220^low^/CD11b^+^ subset (Figures 3(a)–3(c)), suggesting that HDAC activity blockade promoted the migration of B1 cells from the mesenteric lamina into the peritoneal fluid. This hypothesis is corroborated by the fact that 5 days after iHDAC injection in the peritoneal cavity, the mesenteric lamina presented a lower cell density when compared to the control (Figures 3(e) and 3(f)). We highlight the presence of degranulated mast cells observed only in the group that had been injected with iHDAC (Figures 3(A)′ and 3(B)′). Given that mast cells are an important source of IL-5, we suggest that HDAC activity can also modulate the physiology of the peritoneal cavity by also controlling mastocyte behavior. Interestingly, IgM was mostly secreted by B cells from iHDAC-treated mice (Figure 3(g)), suggesting that HDAC activity is necessary for B1 cell behavior and function. However, further experiments need to be performed to clearly address this relationship. Given that AID silencing expression by iHDACs is followed by class-*switch* impairment in B cells (White et al. [63]), AID might mechanistically bridge HDAC activity and B1 cell behavior.

## 3. Conclusion

**Figure 4:**
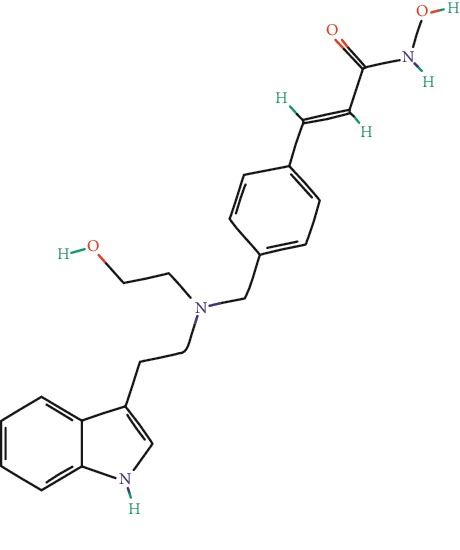
Schematic illustrating the impact of iHDAC on the peritoneal physiology and B1 cell behavior. Under normal conditions (control), the peritoneal cavity is composed of a diverse set of cell types including B lymphocytes (B1 and B2), monocytes, macrophages, and mast cells. The mesenteric lamina presents high cellular density (blue dots). After inhibition of HDAC activity (iHDAC), the mesenteric lamina displays a lower cell density coupled to the increasing amount of B1 cells, degranulated mast cells, and elongated macrophages. In this scenario, iHDAC might emerge as a tractable epigenetic therapy to modulate the physiology of the peritoneal cavity in addition to modulating B1 cells, mast cells, and macrophage biology and behavior.

In this study, we call attention to the importance of the peritoneal cavity that is considered not only as being one of the largest areas involved by a serous membrane but also as a dynamical structure that crosstalks directly with cells of the innate immunity (Figure 4). Indeed, from the point of view of its anatomy, the peritoneal cavity *per se* can be considered as an “autonomous organ” that allows the free passage not only of fluids but also of cells and drugs in and out of milk spots without interfering in the systemic lymphoid structures. These two characteristics place it as an odd structure involved in the generation of a cellular response triggered by antigens and by epigenetic drugs that modulate chromatin status. In this sense, and taking into account that B1 cells are pivotal for autoimmune diseases, iHDAC emerges in this scenario as a putative modulator for this class of disease. Since at least 3 iHDACs are currently approved for use in the clinic, we envisage that further work on the role of iHDACs might be deeply explored in order to shed light on the role of HDAC-dependent epigenetic mechanisms as a promising approach to handle B1 cell behavior's disruption and diseases.

## Figures and Tables

**Table 1:** Main iHDACs used for preclinical and translational studies. The table provides information regarding the name of the iHDAC, the IC_50_, and registered trials found at clinicaltrials.gov in addition to published papers that have used iHDACs to better understand the relevance of HDAC activity in the context of B cell behavior and biology. Chemical structures were obtained from PubChem (https://pubchem.ncbi.nlm.nih.gov/).

iHDAC	IC_50_	Clinical trial identifier	HDAC specificity	Chemical structure	Biologic event	Cell type	Model	Reference
Citarinostat (ACY-241)	2.6 nM (HDAC 6) and 46 nM (HDAC 3)	No trials registered	HDACs 1, 2, 3, and 6	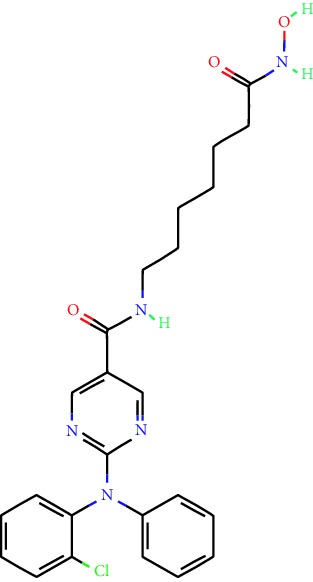	Inhibition of plasma cell myeloma proliferation and survival; cell cycle disruption	MM1, H929, U266	Human	[74]

ACY-738	1.7 nM for HDAC 6 selectively	No trials registered	HDACs 1, 2, 3, and 6	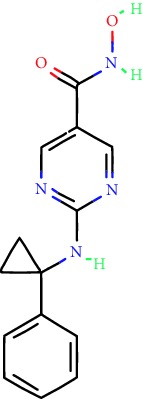	Pre-B cell growth inhibition in lupus disease^∗^	MRL/lpr bone marrow cells	Mouse	[68]

BML-281	0.002 nM (HDAC 6), 271 nM (HDAC 1), 252 nM (HDAC 2), 0.42 nM (HDAC 3), 6851 nM (HDAC 8), 90.7 nM (HDAC 10)	No trials registered	HDAC 6	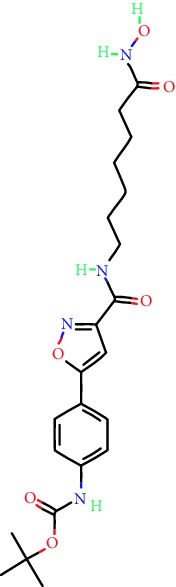	Blocks B cell infiltration in acute colitis^∗^	CD19^+^ B lymphocyte	Mouse	[71]

Dacinostat (LAQ824)	32 nM	No trials registered	Pan iHDAC	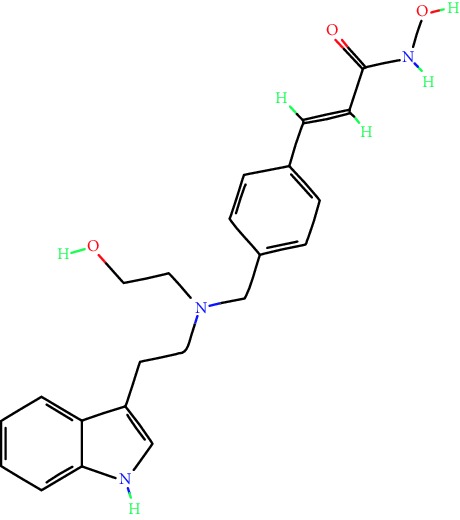	Decreases viability in B-ALL, multiple myeloma, and B lymphoma cells	SEMK RS4;11 SEMK2 697 Namalwa Daudi Ramos MM1S-N OPM2 PMI8226	Human	[75]

Givinostat (ITF2357)	BCR-ABL signaling pathway	No trials registered	Classes I, II	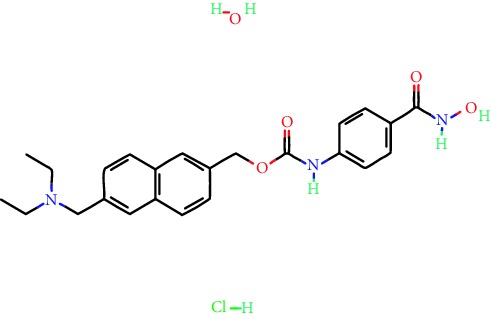	Cell proliferation inhibition and apoptosis induction in chronic myelogenous leukemia, BCR-ABL1-positive and childhood B acute lymphoblastic leukemia	K-562, SUP-B15	Human	[76]

LMK-235	11.9 nM (HDAC 4), 4.2 nM (HDAC 5)	No trials registered	HDACs 4, 5	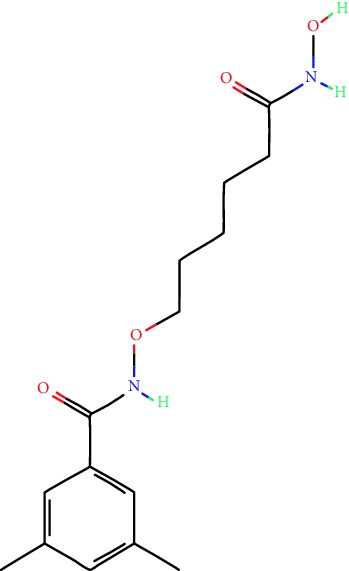	Induces apoptosis and BCLA1 overexpression in diffuse large B cell lymphoma	OCI-Ly10, OCI-Ly3	Human	[77]

Entinostat (MS-275)	0.51 *μ*M (HDAC 1), 1.7 *μ*M (HDAC 3) in cell-free assays	(1) NCT03925428 (2) NCT00098891 (3) NCT00020579	HDACs 1, 3, 4, 6, 8, and 10	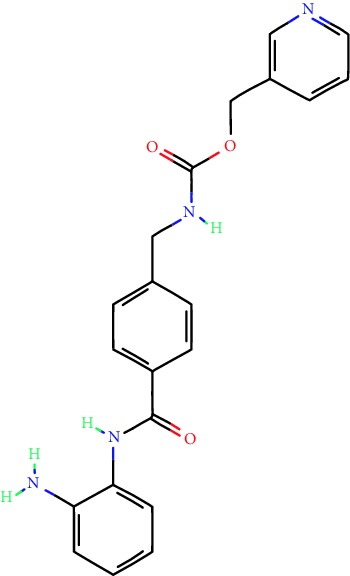	Proliferation inhibition and apoptosis induction; enhancement of DNA damage response in plasma cell myeloma	U266, MM1.R, RPMI8226	Human	[78]
					Dose-/time-dependent cell death, gene expression disruption, and CD20 upregulation in rituximab-sensitive Burkitt lymphoma (BL) and RL (germinal centre B cell)	Raji-4RH, RL-4RH, and U2932-4RH and lymphoma cells derived from patients with untreated or relapsed/refractory B cell lymphoma	Human	[79]
					Decreases cell viability in B-ALL, B-lymphoma, and multiple myeloma cell lines	SEMK2, RS4;11, SEMK2, 697, Namalwa, Daudi, Ramos, MM1S-LN, OPM2, RPMI8226	Human	[75]

Panobinostat (LBH589)	5 nM in a cell-freeassay	(1) NCT01282476 (2) NCT01238692 (3) NCT01523834 (4) NCT00978432 (5) NCT00918333 (6) NCT01261247 (7) NCT00962507 (8) NCT02961816 European Medicines Agency approved for use and FDA accelerated approval for use in multiple myeloma (2015)	Classes I, II, and IV	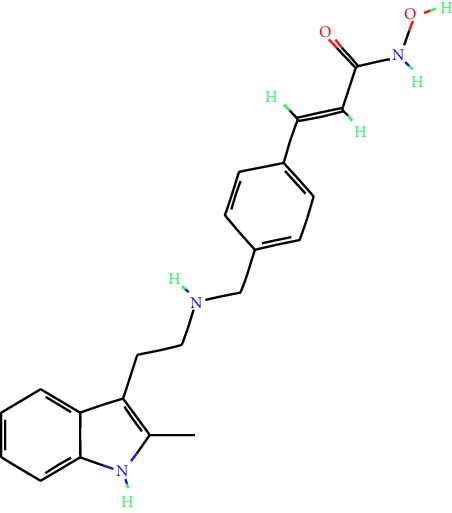	Reduced cell number and viability; delayed division progression; decreases the number of CD138^+^ antibody-secreting cells	B cell B220^+^, CD19^+^, IgM^+^, IgD^+^	Mouse	[69]
					Reduces autoantibody-producing plasma cells^∗^	MRL/lpr mouse autoimmunity	Mouse	[69]
					Primary germinal centre response inhibition	C57BL/6	Mouse	[69]
					Dose-dependent proliferation and tumor growth inhibition; apoptosis induction	CLBL-1 cells	Dog	[80]

RGFP966	0.08 *μ*M in cell-free assay	No trials registered	HDAC 3	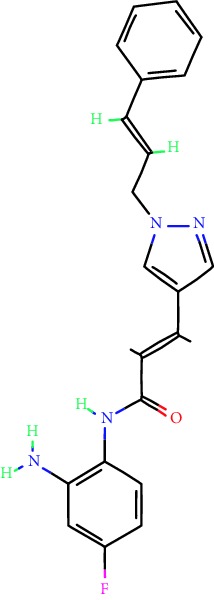	Induces apoptosis, decreases Bcl-2 and Bcl-xL expression. Myc-mediated miR expression	Epstein-Barr virus-related Burkitt lymphoma	Human	[81]
						E*μ*-myc EM330	Mouse	

Ricolinostat (ACY-1215)	5 nM in a cell-free assay. Low activity against HDAC 4/5/7/9/11, sirtuin 1, and sirtuin 2	(1) NCT02091063 (2) NCT02787369	HDAC 6	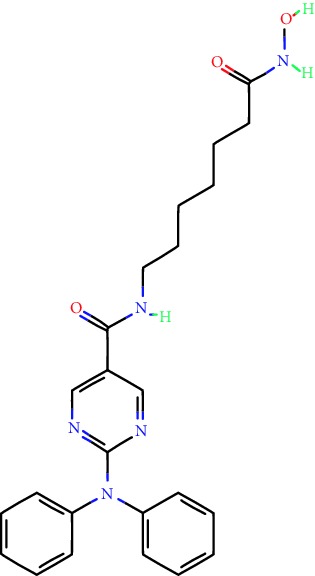	CD20 expression	Peripheral mononuclear cell from CLL patients Raji cells	Human	[82]

Romidepsin (FK228, depsipeptide)	36 nM (HDAC 1), 47 nM (HDAC 2) in cell-free assays	(1) NCT01846390 (2) NCT02281279 (3) NCT00079443 (4) NCT00963274 (5) NCT01897012 (6) NCT02181218 (7) NCT00383565 (8) NCT03432741 (9) NCT01947140 (10) NCT01998035 FDA approved for cutaneous T cell lymphoma (2009)	HDACs 1, 2	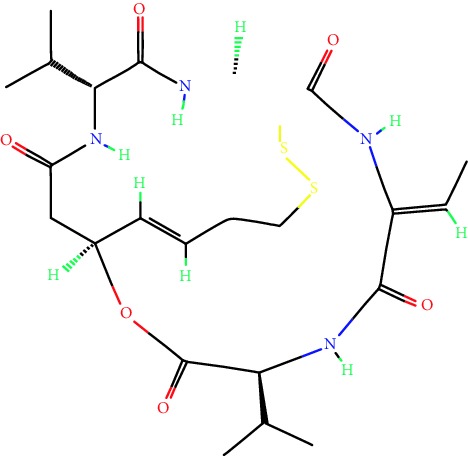	Reduced cell number and viability	B cell B220^+^, CD19^+^, IgM^+^, IgD^+^	Mouse	[69]

Tacedinaline (CI994)	0.9 *μ*M (HDAC 1), 0.9 *μ*M (HDAC 2), 1.2 *μ*M (HDAC 3), >20 *μ*M (HDAC 8)	No trials registered	Class I	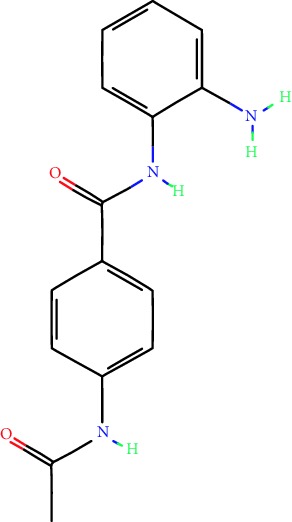	Dose-dependent proliferation inhibition	CLBL-1 cells	Dog	[80]

Trichostatin A (TSA)	1.8 nM in cell-free assays	No trials registered	Pan iHDAC	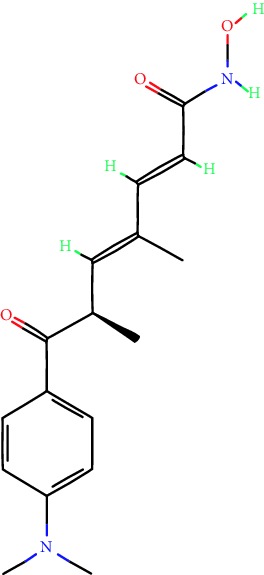	CD20 expression	Raji cells	Human	[82]
					Dose-dependent proliferation inhibition	CLBL-1 cells	Dog	[80]

Tubacin	4 nM in a cell-free assay	No trials registered	HDAC 6	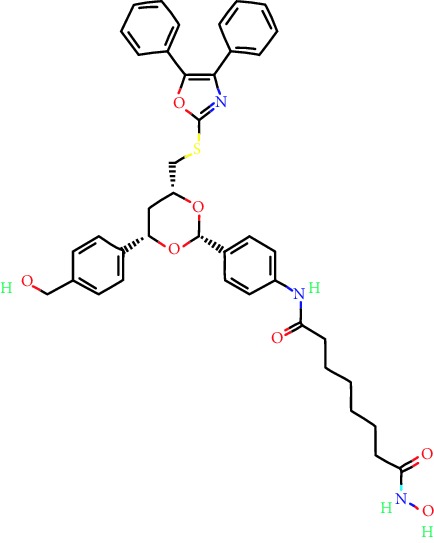	CD20 expression	EBV-positive Burkitt lymphoma EBV-negative Burkitt lymphoma EBV-negative DLBCL cell lines EBV-transformed lymphoblastoid cell lines Peripheral mononuclear cell from CLL patients Raji cells	Human	[82]
					Dose-dependent proliferation inhibition	CLBL-1 cells	Dog	[80]

Tubastatin A	15 nM in a cell-free assay	No trials registered	HDAC 6	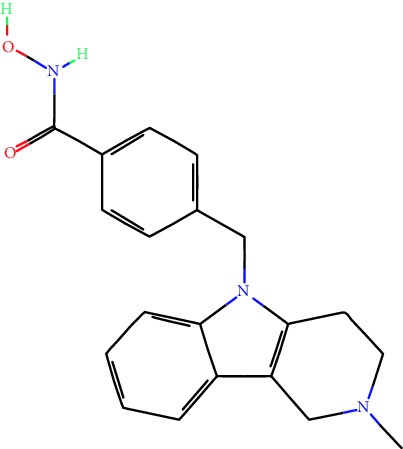	CD20 expression	Raji cells	Human	[82]

Vorinostat (SAHA, MK0683)	10 nM in a cell-free assay	(1) NCT00097929 (2) NCT00764517 (3) NCT02589145 (4) NCT03150329 (5) NCT00667615 (6) NCT00703664 (7) NCT01193842 (8) NCT00972478 (9) NCT00875056 (10) NCT01567709 (11) NCT01120834 (12) NCT01276717 (13) NCT00992446 (14) NCT00499811 (15) NCT01116154 (16) NCT01789255 (17) NCT01500538 (18) NCT00601718 (19) NCT00275080 (20) NCT03259503 (21) NCT00253630 (22) NCT03842696 (23) NCT00791011 (24) NCT00994500 (25) NCT00217412 (26) NCT00837174 (27) NCT00720876 (28) NCT00005634 (29) NCT00918723 FDA approved for cutaneous T lymphoma (2006)	Classes I, II, and IV	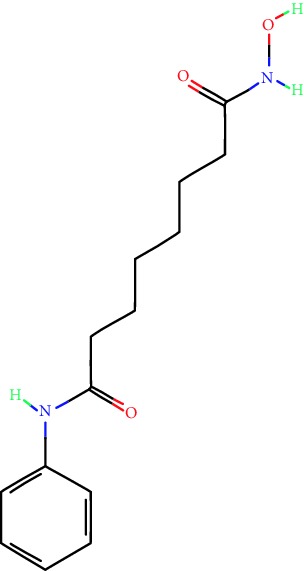	CD20 expression	Raji cells	Human	[82]
					Reduced cell number and viability; delayed division progression; decreases the number of CD138^+^ antibody-secreting cells	B cell B220^+^, CD19^+^, IgM^+^, IgD^+^	Mouse	[69]
					Dose-dependent proliferation inhibition	CLBL-1 cells	Dog	[80]
					Cell viability decrease, apoptosis induction	Raji, Raji-4RH, RL-4RH, RL, and patient primary tumors	Human	[83]
					Enhances apoptosis mediated by kinase inhibitors that affect BCR signaling and gene expression disruption in mantle cell lymphoma	Jeko-1, Mino	Human	[84]
					Induces cell death in rituximab-sensitive Burkitt lymphoma (BL) and RL (germinal centre B cell)	Lymphoma cells derived from patients with untreated or relapsed/refractory B cell lymphoma	Human	[79]

Scriptaid	9 *μ*M (Ishikawa endometrial cancer cell line) and 55 *μ*M (SK-OV-3 ovarian cancer cell line)	No trials registered	Pan iHDAC	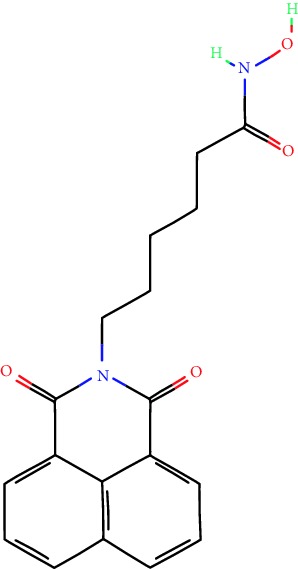	CD20 expression	Raji cells	Human	[82]
					Dose-dependent proliferation inhibition	CLBL-1 cells	Dog	[80]

Suberohydroxamic acid (SBHA)	0.25 *μ*M (HDAC 1), 0.3 *μ*M (HDAC 3)	No trials registered	HDACs 1 and 3	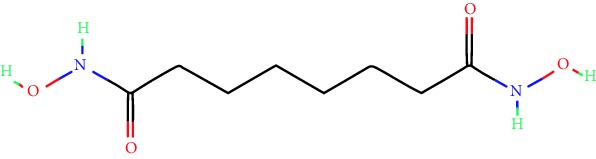	Dose-dependent proliferation inhibition	CLBL-1 cells	Dog	[80]
					Src tyrosine kinase Gardner-Rasheed feline sarcoma viral (v-FGR) oncogene homolog (FGR) mediates SAHA resistance	BL-2, BL-41, BL-70, Blue-1, CA-46, Daudi, DG-75, DND-39, Namalwa, Raji (Burkitt lymphoma), Carnaval, Granta-452, HBL-1, HT, Kis-1, OCI-Ly1, OCI-Ly2, OCI-Ly3, OCI-Ly7, OCI-Ly10, SU-DHL-4, SU-DHL-6, TMD8, U2932, WSU-DLCL2, WSU-FSCCL (diffuse large B cell lymphoma)	Human	[85]

Valproic acid	Selectively induces proteasomal degradation of HDAC 2	(1) NCT01622439 (2) NCT00109824 (3) NCT02144623 Approved for use in the treatment of epilepsy	Pan iHDAC	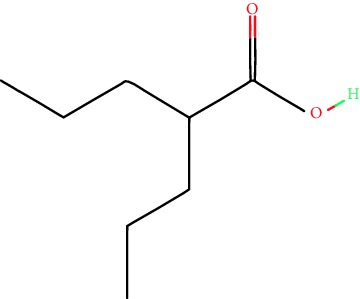	Class-switch DNA recombination (CSR) and plasma cell differentiation	C57BL/6J	Mouse	[86, 87]
					CD20 expression	CLL cell line CLL patients	Human	[88]

WT161	8.35 nM (HDAC 1), 15.4 nM (HDAC 2), 0.4 nM (HADC6)	No trials registered	HDACs 1, 2, and 6	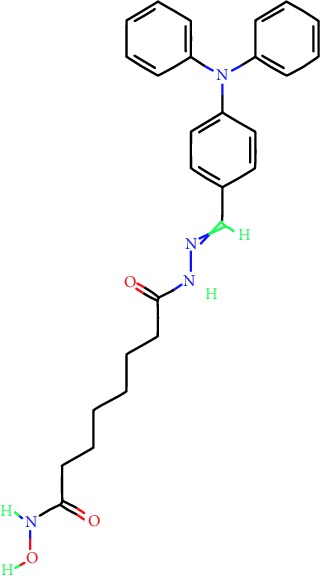	Decreases cell viability in B-ALL, B lymphoma, and multiple myeloma cell lines	SEMK2, RS4;11, SEMK2, 697	Human	[75]

**Table 2:** Studies that have used molecular tools to explore the role of specific HDACs in B cell biology and behavior. The table summarizes published papers that have used molecular approaches and/or knockout models to better understand the relevance of a particular HDAC in the context of B cell behavior and biology.

	Target	Effect	Cell line	Model	Reference
HDAC 1	Myc	Suppresses tumor growth in E*μ*-myc-driven B cell lymphoma	HDAC 1 knockout mice	Mouse	[89]
	p21 upregulation	Cell growth inhibition and apoptosis induction	shRNA RS4;11 SEMK2 cells	Human	[75]
	No defined molecular target	Increased cell death	shRNA RS4;11, REH, 697, and SEMK2 cells		

HDAC 2	Myc	Suppresses tumor growth in E*μ*-myc-driven B cell lymphoma	HDAC 1 knockout mice	Mouse	[89]
	p21 upregulation	Cell growth inhibition and apoptosis induction	shRNA RS4;11 SEMK2 cells	Human	[75]
	No defined molecular target	Increased cell death	shRNA RS4;11, REH, 697, and SEMK2 cells		

HDAC 3	MHC class II gene expression	Blocks lymphoma growth	Lymphoma cell lines (OCI-Ly7, MD901, OCI-Ly18, OZ, and RIVA)	Human	[90]
	p21 and H2AX increased levels	Increased cell death	shRNA RS4;11, REH, 697, and SEMK2 cells	Human	[75]

HDAC 4	miR-155	Upregulation decreases proliferation and clonogenic potential and increases apoptosis	E*μ*-miR-155 transgenic mouse model	Mouse	[91]

HDAC 6	No defined molecular target	HDAC 6 is upregulated	MRL/lpr mouse autoimmunity Bone marrow and splenic B cells	Mouse	[68]
	Activation of ECM signaling	Differentially expressed in human diffuse large B cell lymphoma tissues; correlated with poor prognosis	Patient sample; NuDUL-1	Human	[92]

HDAC 7	Itgam and CD69 promoter	Pro-B to pre-B cell transition blockade; severe lymphopenia in peripheral organs	Pro-B cells	Mouse	[93]

HDAC 9	No defined molecular target	HDAC 9 is upregulated	MRL/lpr mouse autoimmunity Bone marrow B cells	Mouse	[68]
	BCL6 p53	Constitutive expression induces lymphomagenesis	E*μ*-HDAC 9 transgenic mouse	Mouse	[94]

HDAC 10	No defined molecular target	HDAC 10 is downregulated	MRL/lpr mouse autoimmunity Bone marrow B cells	Mouse	[68]
		HDAC 10 is upregulated	MRL/lpr mouse autoimmunity Splenic B cells	Mouse	[68]

HDAC 11	IL-10	Allergy (rhinitis)	Patients	Human	[95]
